# Public perceptions of multiple risks during the COVID-19 pandemic in Italy and Sweden

**DOI:** 10.1038/s41597-020-00778-7

**Published:** 2020-12-10

**Authors:** Elena Mondino, Giuliano Di Baldassarre, Johanna Mård, Elena Ridolfi, Maria Rusca

**Affiliations:** 1 Centre of Natural Hazards and Disaster Science, Uppsala, Sweden; 2 Department of Earth Sciences, Uppsala University, Uppsala, Sweden

**Keywords:** Natural hazards, Psychology and behaviour, Interdisciplinary studies

## Abstract

Knowing how people perceive multiple risks is essential to the management and promotion of public health and safety. Here we present a dataset based on a survey (N = 4,154) of public risk perception in Italy and Sweden during the COVID-19 pandemic. Both countries were heavily affected by the first wave of infections in Spring 2020, but their governmental responses were very different. As such, the dataset offers unique opportunities to investigate the role of governmental responses in shaping public risk perception. In addition to epidemics, the survey considered indirect effects of COVID-19 (domestic violence, economic crises), as well as global (climate change) and local (wildfires, floods, droughts, earthquakes, terror attacks) threats. The survey examines perceived likelihoods and impacts, individual and authorities’ preparedness and knowledge, and socio-demographic indicators. Hence, the resulting dataset has the potential to enable a plethora of analyses on social, cultural and institutional factors influencing the way in which people perceive risk.

## Abstract (summary)

**Table:** 

Measurement(s)	risk perception
Technology Type(s)	survey method
Factor Type(s)	experience • preparedness • knowledge • age • sex • education • income • occupation • political orientation • region of residence
Sample Characteristic - Organism	Homo sapiens
Sample Characteristic - Location	Italy • Sweden

Machine-accessible metadata file describing the reported data: 10.6084/m9.figshare.13213832

## Background & Summary

Risk is often seen as a combination of hazard, exposure, and vulnerability^1^. While some individuals might be unaware of the potential occurrence of a given hazard (e.g. epidemics, earthquakes or floods), others might misjudge their level of exposure, vulnerability, or both. Knowing how people perceive multiple risks, and how public perceptions are shaped by the occurrence of major crises^2–4^, is key in: (a) detecting windows of opportunity for policy change^5,6^, (b) improving risk management strategies^7,8^, and (c) supporting communication between decision-makers and the general public^9^.

This paper presents a new dataset that provides unique opportunities to investigate public risk perception under the COVID-19 pandemic. We explore public perceptions of risk in Italy and Sweden around nine threats: epidemics, floods, droughts, earthquakes, wildfires, terror attacks, domestic violence, economic crises, and climate change. A total of 4,154 participants (N_ITA_ = 2,033, N_SWE_ = 2,121) have been surveyed online and the results are assembled in this dataset. The survey was performed in the period 5–19 August 2020 and will be repeated in November 2020 and August 2021.

Italy and Sweden were selected due to similarities and differences allowing for numerous types of comparative analyses. Both countries have been severely affected by the first wave of COVID-19 and, at the time of the survey, they suffered a similar number of COVID-19 related fatalities^10^: 35,412 in Italy (586 per 1 million inhabitants) and 5,812 in Sweden (575 per 1 million inhabitants). Moreover, while a new wave of infections took speed in many European countries^11^, the spread of the virus in Italy and Sweden had low rates in August 2020, with less than 30 daily new cases per one million inhabitants^10^.

Yet, governmental responses to the first wave of COVID-19 infections in Spring 2020 have been radically different^12^. Italy was the first European country to introduce a national lockdown in early March 2020. The Italian Government issued various decrees over time to introduce (or lift) strict measures^13^, such as closing schools for more than six months. In contrast, Sweden emerged as an international outlier because of its less stringent measures for coping with the COVID-19 pandemic. The Swedish approach, driven primarily by the Public Health Agency of Sweden, has been based on voluntary compliance with a set of regulations and recommendations^14^.

Similarities and differences in how people in Sweden and Italy experienced the COVID-19 pandemic provide an unprecedented opportunity to explore public perceptions of multiple risks under the common pandemic threat. Hence, the information collected in this dataset can enable the study of how e.g. different governmental responses^12^ influence the way in which people perceive epidemic risk. In fact, within game theory, risk perception has been found to particularly influence epidemics’ peaks^15^. This is relevant as epidemic peaks, such as the first COVID-19 infection wave, can have a disastrous impact on healthcare capacity, and, therefore, on the unfolding of the epidemic itself.

The rationale for the selection of multiple threats was twofold: global relevance and local impacts in the surveyed countries. Besides the COVID-19 pandemic, the climate crisis remains a major global threat. Thus, we survey perceptions about climate change to put quantitative results into perspective (e.g. how do concerns about epidemics compare with those about climate change?). This is particularly relevant because of the high number of young people who took part in the *Fridays for Future* strikes in both Italy^16^ and Sweden^17^ in the period prior to the pandemic (late 2019 – early 2020). Such a large participation is evidence of a strong concern for the global climate crisis and raises questions of intergenerational risk perception that can be further investigated with this dataset.

The other threats included in the survey encompass those that have impacted one or both countries with different levels of severity and frequency over the last decade. Catastrophic wildfires^18^ and a deadly terrorist attack^19^ recently occurred in Sweden, while many fatalities or economic losses have been caused by earthquakes, floods and droughts in Italy over the past decade^20^. We also included economic crises and domestic violence as they are potential (indirect) effects of the COVID-19 pandemic and governmental responses. The inclusion of these additional threats has the potential to support future studies aiming to e.g. unravel how experience, media coverage, trust, inequality or social capital shape public perceptions of risk.

## Methods

To provide exhaustive information on the survey to facilitate reproducibility, we follow the Checklist for Reporting Results of Internet E-Surveys (CHERRIES)^21^.

### Survey design

The survey was designed following previous studies^8,22,23^. It covers people’s perceptions on five main factors: likelihood of the phenomenon to occur, potential impact of the phenomenon, preparedness to face the phenomenon, knowledge around the phenomenon, and experience with the phenomenon. The questions used to investigate these factors are listed in Table 1. Each question was asked in relation to epidemics, floods, droughts, wildfires, earthquakes, terror attacks, domestic violence, economic crises, and climate change.

**Table 1:** Survey questions and the related available answers.

Variable	Question	Available answers
Likelihood	How likely do you think it is that you are directly involved in the following phenomena*?	On a scale from 1, “Very unlikely” to 5, “Very likely”, or “I don’t know”
Potential impact		
On the respondent	In case you are involved, how much damage do you think the following phenomena* can cause to yourself?	On a scale from 1, “No damage” to 5, “Severe damage”, or “I don’t know”
On other people in the country	In case they would occur in [country**], how much damage do you think the following phenomena* can cause to others in the country?	On a scale from 1, “No damage” to 5, “Severe damage”, or “I don’t know”
Preparedness		
Of the respondent	How prepared do you think you are to face the following phenomena*?	On a scale from 1, “Not at all prepared” to 5, “Highly prepared”, or “I don’t know”
Of the authorities in the country	How prepared do you think the responsible authorities in [country**] are to face the following phenomena?	On a scale from 1, “Not at all prepared” to 5, “Highly prepared”, or “I don’t know”
Knowledge		
Of the respondent	How knowledgeable are you about the following phenomena?	On a scale from 1, “Not at all knowledgeable” to 5, “Highly knowledgeable”, or “I don’t know”
Of the authorities in the country	How knowledgeable do you think the responsible authorities in [country**] are about the following phenomena*?	On a scale from 1, “Not at all knowledgeable” to 5, “Highly knowledgeable”, or “I don’t know”
Experience	Have you ever been directly involved in any of the following phenomena*, in your country or abroad?	1, Yes 2, No or “I don’t know”

*Epidemics, floods, droughts, wildfires, earthquakes, terror attacks, domestic violence, economic crises,

climate change.

**Italy, Sweden.

In addition to the questions in Table 1, the respondents were also asked to provide socio-demographic information, including their age, gender, maximum level of education achieved, income, type of employment (if any), and political orientation. The survey contained a total of 77 items, as the age and gender of respondents was known in advance. The full survey form can be found in the supplementary material and in the dataset repository^24^.

### Survey administration

The survey was targeted to individuals living in Italy and in Sweden. The two samples were drawn from two existing survey panels, one in Italy and one in Sweden. Both panels were set up by Kantar Sifo^25^, one of Sweden’s major marketing research companies, and they include around 100,000 individuals in each country. Each panel consists of randomly recruited individuals, from 16 years and up, and is representative of the country’s population. The participants are gradually replaced, and the panel is filled with new respondents to prevent the participants from becoming experts or too accustomed to the survey methodology. Panelist collect points for each survey they agree to fill in, and the points can then be turned into various types of rewards (e.g. movie tickets, or gift cards). Concerning this specific survey, the panelists were contacted via e-mail, and if they did not respond the first time, up to two reminders were sent during the survey period (5–19 August 2020). In the email, panelists were informed on the estimated length of the survey (around 12 minutes) and were directed to the Privacy Policy, where they could also find information on data storage. To avoid potential biases, information about the investigator and the purpose of the study were not communicated to the panelists. The link to the online survey was sent via e-mail, and only to panelists who accepted to fill in the survey (closed survey). Each question in the form allowed for “I do not know” or “I’d rather not say” options. At the end of the survey, respondents could go back to previous questions to check or edit their answers. Each respondent could fill in the survey only once. As for the response rate, 27% of contacted panelists eventually completed the survey. The completion rate, i.e. the ratio of panelists who finished and submitted the survey and panelists who agreed to participate, was 86.2% in Italy and 89.5% in Sweden. All questionnaires that were submitted are included in the dataset, regardless of their completeness). All responses were automatically captured by the software. The survey was administered only to adults, i.e. over 18 years. The respondents did not have the option of choosing the language, which was Italian for respondents in Italy, and Swedish for respondents in Sweden. The two samples are representative of the two countries’ population in terms of age and gender.

### Ethical approval

The research protocol for this study was approved by the *Italian Research Ethics and Bioethics Committee* (protocol 0043071/2019) and the *Swedish Ethical Review Authority* (Dnr 2019-03242). All procedures performed in the study were in accordance with the ethical standards set by the European Union under Horizon 2020 (EU General Data Protection Regulation and FAIR Data Management). Participants were advised that participation is voluntary and that the outcomes are anonymous. The research protocol did not encompass collection of privacy-sensitive and personally identifiable information (PII) data. Informed consent was obtained from all participants involved in the study.

## Data Records

The dataset resulting from the online survey comes in a CSV format and is stored open access on Zenodo^24^. Each row represents one respondent and each column represents a variable (i.e. one column for each survey question for each phenomenon, and one column for each socio-demographic variable). The cell value represents the answer that the respondent gave to the question. For instance, if the answer had to be given on a scale from 1 (minimum) to 5 (maximum), then the cell value is either 1, 2, 3, 4, 5, or the respective code in case the respondent did not select any option or selected “I do not know”/“I’d rather not say”. The dataset comes with a metadata file (.xlsx) that provides detailed information on the variables and the coding of the answers. The survey forms in Italian and in Swedish are stored in the same repository, along with the English translation.

A total of 2,033 respondents completed the survey in Italy, and 2,121 in Sweden. The dataset is divided into two samples, representing the two countries. Each sample is then divided into two subsamples, one representing the capital’s region (Rome – Lazio, N = 1,007; Stockholm – Stockholm county, N = 1,036), and one representing the rest of the country. The distribution of the two samples – 50% from the capital’s region and 50% from the rest of the country – allows for additional studies on the spatial distribution of risk perception. For instance, the risk of terror attacks might be perceived differently in capital cities, compared to the rest of the country.

**Fig. 1:**
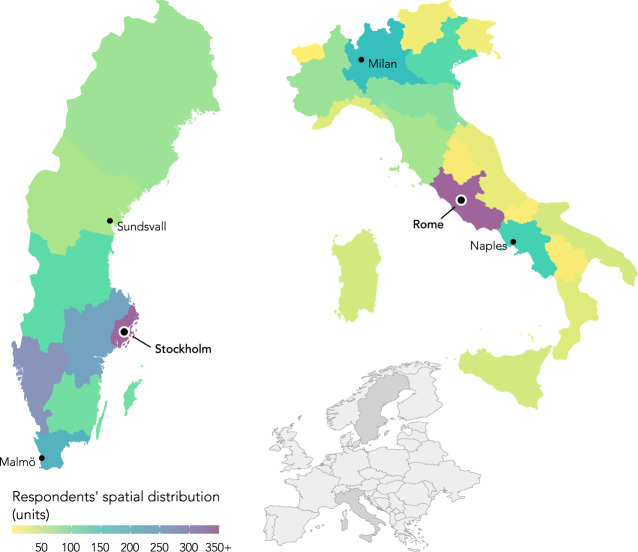
Spatial distribution of respondents in Sweden and Italy.

The sample collected in Italy is slightly younger (M = 49.16, SD = 13.70, min = 18, max = 79), 47% of respondents are males and 53% are females. In comparison, the one collected in Sweden is slightly older (M = 49.78, SD = 15.75, min = 19, max = 79), 52% of respondents are males and 48% are females. Figure 1 shows the spatial distribution of the respondents in the two countries, which is representative of the country’s population. Thus, there are more respondents in the most populous regions. Response rates to each of the questions, per country, are listed in Table S1.

## Technical Validation

Before conducting the survey, we assessed its feasibility and validity^26^. The survey questions were designed by the authors of this paper, who have previous experience with survey design on risk perception and preparedness^8,27–29^. The survey questions were designed on the basis of published studies^8,22,23^, so that results could be comparable with prior surveys and face validity of the survey could be ensured (i.e. whether the questions appear reasonable to acquire the necessary data). As those who designed the survey are primarily experts in the field of natural hazards, the survey went through a round of pre-testing to ensure its internal validity, i.e. making sure that the questions posed serve the purpose of the survey and that there is no potential for misunderstandings (as some concepts may be obvious to the researcher, but not to the respondent). The preliminary survey was thus administered to a total of 12 lay people in Italy and 6 lay people in Sweden, and the respondents were asked whether the questions were clear and how they interpreted them. This step ensured that the respondents interpreted the questions as expected. The final survey form was then reviewed by Kantar Sifo^25^, which then was appointed to undertake data collection.

The use of an external professional research firm ensured the validity and reliability of the survey thanks to several levels of quality control before, during, and after data collection. The potential for human error when it comes to data entry is expected to be minimal, as responses to the survey were automatically collected in the dataset through the online survey system. As a matter of fact, we did not find any systematic problem due to response-bias connected to the type of phenomenon, or any other variable, as shown in Table S1. Errors in survey response can be assumed to be random, and not correlated with any demographic or personal characteristics.

## Usage Notes

This dataset provides a plethora of opportunities to explore multiple facets related to numerous public perceptions of risk, for both natural hazards and man-made threats. It also provides insights into public trust in national authorities, preparedness to face different types of threats, and levels of risk awareness. Moreover, the contrasting nature of the Swedish and Italian contexts, which was recently highlighted by the different response to the first wave of the COVID-19 pandemic, allows for insightful comparative studies.

We present a non-exhaustive list of scientific questions that can be addressed with the help of this dataset:

- How prepared to face an epidemic do citizens feel in the two countries? How much knowledge of the epidemic threat do they believe they have?
- What are the most concerning threats for the citizens in the two countries? How is public perception influenced by perceived individual and authorities’ knowledge?
- How are people’s concerns affected by their experience? These questions can shed light on potential discrepancies between perceived and actual risk, especially given the spatial distribution of the samples (Fig. 1).
- How much do citizens in the two countries trust the responsible authorities when it comes to risk management? How is this connected to perceived individual and authorities’ preparedness?
- Do the socio-demographic variables show trends connected to e.g. age, or education?

The above list is just an example of the number of questions this dataset has the potential to address, alone as an independent dataset or together with other pre-existing data. Indeed, this dataset could gain even deeper value if coupled with other relevant datasets that were recently published, such as the global analysis of COVID-19 risk perception by Dryhurst *et al*.^30^, performed in Spring 2020 with smaller national samples but including more countries, or the dataset on government interventions in response to COVID-19 by Desvars-Larrive *et al*.^12^. Such valuable information does not only have scientific relevance, but also has the potential to inform policy makers for developing and updating risk management policies, while improving risk communication campaigns.

### Illustrative exploratory analysis

**Fig. 2:**
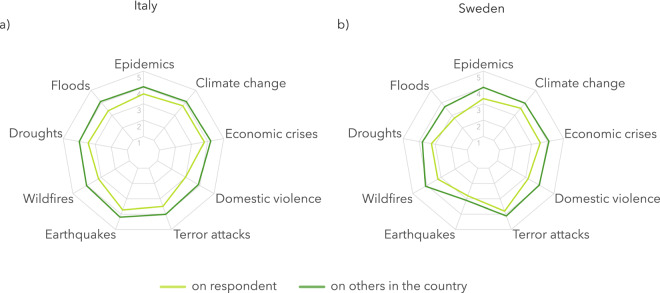
Average values in terms of impact on respondent and impact on others in the country for each of the nine phenomena, by country.

To show the potential of the dataset, we present a simple exploratory analysis of the differences between the two samples. One of the interesting results that emerged from the data is that both countries show an optimistic bias in terms of the phenomenon’s impact (Fig. 2a,b). The optimistic bias is the tendency for people to report that they are *less likely than others* to experience negative events^31^. In both countries, respondents show an optimistic bias when it comes to floods (*Χ*^2^_ITA_ = 10.48, p < 0.05; *Χ*^2^_SWE_ = 16.52, p < 0.01), wildfires (*Χ*^2^_ITA_ = 12.08, p < 0.01; *Χ*^2^_SWE_ = 15.80, p < 0.01), and domestic violence (*Χ*^2^_ITA_ = 12.00, p < 0.05; *Χ*^2^_SWE_ = 12.40, p < 0.01). Respondents in Sweden also show an optimistic bias when it comes to epidemics (*Χ*^2^_SWE_ = 11.85, p < 0.05).

**Fig. 3:**
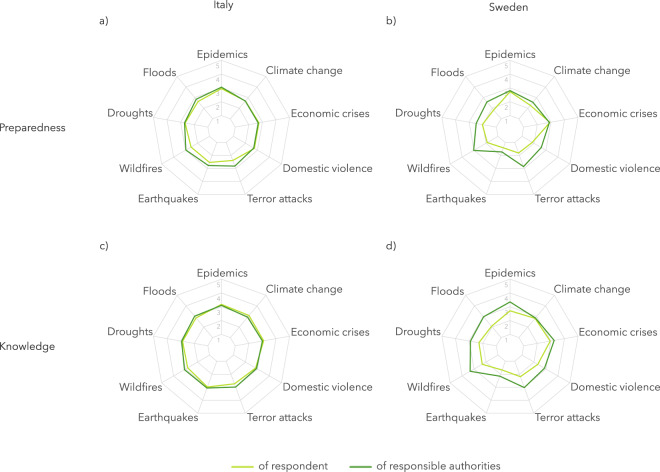
Average values in terms of preparedness and knowledge for each of the nine phenomena, by country.

Additionally, respondents in Italy feel as prepared and as knowledgeable as the responsible authorities for all of the phenomena (as shown in Fig. 3a,c), with no statistically significant difference to be noted. On the contrary, respondents in Sweden tend to perceive their preparedness and knowledge as lower, compared to that of the responsible authorities (see Table 2, and Fig. 3b,d). These results could potentially shed light on country-level differences when it comes to trust in the authorities responsible for risk management, safety and public health.

**Table 2:** Χ^2^ test results on differences in respondents’ perception between respondent’s versus authorities’ preparedness and knowledge, in each country.

	Ep	Fl	Dr	Wf	Eq	TA	DV	EC	CC
**Preparedness**									
Individual vs authorities ITA	0.72	1.20	0.40	4.60	1.50	2.54	0.77	0.18	0.20
Individual vs authorities SWE	0.38	19.84***	7.65*	26.07***	6.07	23.84***	24.64***	0.85	2.96
**Knowledge**									
Individual vs authorities ITA	0.24	0.66	0.39	2.01	0.22	0.88	0.83	0.21	0.73
Individual vs authorities SWE	9.64**	18.77***	9.64**	20.42***	5.69	14.10***	9.76**	2.66	0.05

Ep = epidemics, Fl = floods, Dr = droughts, Wf = wildfires, Eq = earthquakes, TA = terror attacks, DV = domestic violence, EC = economic crises, CC = climate change.

***p < 0.01, **p < 0.05, *p < 0.1.

## Supplementary information

Supplementary material

Table S1

## Data Availability

All code for reading, processing and graphically representing the data is freely available at https://github.com/elenamondino/nationwide_survey. Information on the packages used are listed in the same repository.
