# Supplementary material for: Public perceptions of multiple risks during the COVID-19 pandemic in Italy and Sweden
Source: Sci Data. 2020 Dec 10;7:434. doi: 10.1038/s41597-020-00778-7 (PMC7729954; doi:10.1038/s41597-020-00778-7)
Supplement: Supplementary file 1 — Supplementary material [file 41597_2020_778_MOESM1_ESM.pdf]

English translation of the survey form

Q001 - Q001: Matrix

How likely do you think it is that you are directly involved in the following phenomena?

	1 Not at all likely	2	3	4	5 Very likely	I don’t know *Fixed *Exclusive
Epidemics	❍	❍	❍	❍	❍	❍
Floods	❍	❍	❍	❍	❍	❍
Droughts	❍	❍	❍	❍	❍	❍
Wildfires	❍	❍	❍	❍	❍	❍
Earthquakes	❍	❍	❍	❍	❍	❍
Terror attacks	❍	❍	❍	❍	❍	❍
Domestic violence	❍	❍	❍	❍	❍	❍
Economic crises	❍	❍	❍	❍	❍	❍
Climate change	❍	❍	❍	❍	❍	❍

Q002 - Q002: Matrix

In case you are directly involved, how much damage do you think the following phenomena can cause to you?

	1 No damage	2	3	4	5 Severe damage	I don’t know *Fixed *Exclusive
Epidemics	❍	❍	❍	❍	❍	❍
Floods	❍	❍	❍	❍	❍	❍
Droughts	❍	❍	❍	❍	❍	❍
Wildfires	❍	❍	❍	❍	❍	❍
Earthquakes	❍	❍	❍	❍	❍	❍
Terror attacks	❍	❍	❍	❍	❍	❍
Domestic violence	❍	❍	❍	❍	❍	❍
Economic crises	❍	❍	❍	❍	❍	❍
Climate change	❍	❍	❍	❍	❍	❍

**Q003 - Q003:**	**Matrix**

In case they occur in [country], how much damage do you think the following phenomena can cause to others living in [country]?

	1 No damage	2	3	4	5 Severe damage	I don’t know *Fixed *Exclusive
Epidemics	❍	❍	❍	❍	❍	❍
Floods	❍	❍	❍	❍	❍	❍
Droughts	❍	❍	❍	❍	❍	❍
Wildfires	❍	❍	❍	❍	❍	❍
Earthquakes	❍	❍	❍	❍	❍	❍
Terror attacks	❍	❍	❍	❍	❍	❍
Domestic violence	❍	❍	❍	❍	❍	❍
Economic crises	❍	❍	❍	❍	❍	❍
Climate change	❍	❍	❍	❍	❍	❍

**Q004 - Q004:**	**Matrix**

How prepared do you think the responsible authorities in [country] are to face the following phenomena?

	1 Not at all prepared	2	3	4	5 Highly prepared	I don’t know *Fixed *Exclusive
Epidemics	❍	❍	❍	❍	❍	❍
Floods	❍	❍	❍	❍	❍	❍
Droughts	❍	❍	❍	❍	❍	❍
Wildfires	❍	❍	❍	❍	❍	❍
Earthquakes	❍	❍	❍	❍	❍	❍
Terror attacks	❍	❍	❍	❍	❍	❍
Domestic violence	❍	❍	❍	❍	❍	❍
Economic crises	❍	❍	❍	❍	❍	❍
Climate change	❍	❍	❍	❍	❍	❍

Q005 - Q005: **Matrix**

In case you are directly involved, how prepared do you think you are to face the following phenomena?

	1 Not at all prepared	2	3	4	5 Highly prepared	I don’t know *Fixed *Exclusive
Epidemics	❍	❍	❍	❍	❍	❍
Floods	❍	❍	❍	❍	❍	❍
Droughts	❍	❍	❍	❍	❍	❍
Wildfires	❍	❍	❍	❍	❍	❍
Earthquakes	❍	❍	❍	❍	❍	❍
Terror attacks	❍	❍	❍	❍	❍	❍
Domestic violence	❍	❍	❍	❍	❍	❍
Economic crises	❍	❍	❍	❍	❍	❍
Climate change	❍	❍	❍	❍	❍	❍

Q006 - Q006: **Matrix**

How knowledgeable do you think the responsible authorities in [country] are on the following phenomena?

	1 Not at all knowledgeable	2	3	4	5 Highly knowledgeable	I don’t know *Fixed *Exclusive
Epidemics	❍	❍	❍	❍	❍	❍
Floods	❍	❍	❍	❍	❍	❍
Droughts	❍	❍	❍	❍	❍	❍
Wildfires	❍	❍	❍	❍	❍	❍
Earthquakes	❍	❍	❍	❍	❍	❍
Terror attacks	❍	❍	❍	❍	❍	❍
Domestic violence	❍	❍	❍	❍	❍	❍
Economic crises	❍	❍	❍	❍	❍	❍
Climate change	❍	❍	❍	❍	❍	❍

Q007 - Q007: Matrix

How knowledgeable are you on the following phenomena?

	1 Not at all knowledgeable	2	3	4	5 Highly knowledgeable	I don’t know **Fixed *Exclusive*
Epidemics	❍	❍	❍	❍	❍	❍
Floods	❍	❍	❍	❍	❍	❍
Droughts	❍	❍	❍	❍	❍	❍
Wildfires	❍	❍	❍	❍	❍	❍
Earthquakes	❍	❍	❍	❍	❍	❍
Terror attacks	❍	❍	❍	❍	❍	❍
Domestic violence	❍	❍	❍	❍	❍	❍
Economic crises	❍	❍	❍	❍	❍	❍
Climate change	❍	❍	❍	❍	❍	❍

Q008 - Q008: Matrix

Have you ever been directly involved in the following phenomena, in [country] or abroad?

	Yes	No	I don’t know **Fixed *Exclusive*
Epidemics	❍	❍	❍
Floods	❍	❍	❍
Droughts	❍	❍	❍
Wildfires	❍	❍	❍
Earthquakes	❍	❍	❍
Terror attacks	❍	❍	❍
Domestic violence	❍	❍	❍
Economic crises	❍	❍	❍
Climate change	❍	❍	❍

Q009 - Q009: Single coded

What is the highest level of education you achieved?

1	Primary school
2	Lower secondary school
3	Professional high-school
4	Upper secondary school/high-school
5	University degree or higher
997	I’d rather not say **Fixed *Exclusive*

Q010 - Q010: **Single coded**

To satisfy your family’s needs, your household income is:

1	1 Insufficient
2	2
3	3
4	4
5	5 More than sufficient
997	I’d rather not say **Fixed *Exclusive*

Q011 - Q011: **Single coded**

Do you have a job?

1	Yes
2	No
997	I’d rather not say **Fixed *Exclusive*

Ask only if **Q011 - Q011**,1

Q012 - Q012: **Single coded**

Which of the following categories best represent the sector in which you are employed?

1	Industry, technology, production
2	IT & telecommunications
3	Media e communication
4	Healthcare
5	Construction and real estate
6	Trade and commerce
7	Transportation and logistics
8	Hotel and restaurants industry
9	Organisations and associations
10	Bank, finance and insurance
11	Other consultancy services
12	Public administration and defense
13	School and education (kindergarten to high-school)
14	Academia and research (university)
15	Culture, leisure, and free time
16	Energy and environment
17	Agriculture, silvicolture and fishery
18	Other services
996	Other category **Fixed*
997	I’d rather not say **Fixed *Exclusive*

Q013 - Q013: **Single coded**

It is said that political opinions can be placed on a left-right scale. Where would you place yourself on such a scale?

1	Left
2	Centre-left
3	Centre
4	Centre-right
5	Right
997	I’d rather not say **Fixed *Exclusive*
