# Supplementary material for: Public perceptions of multiple risks during the COVID-19 pandemic in Italy and Sweden
Source: Sci Data. 2020 Dec 10;7:434. doi: 10.1038/s41597-020-00778-7 (PMC7729954; doi:10.1038/s41597-020-00778-7)
Supplement: Supplementary file 2 — Table S1 [file 41597_2020_778_MOESM2_ESM.docx]

Table 1. Response rate to the survey questions, divided by country.

|  |  | **Response Rate (%)** | |
| --- | --- | --- | --- |
| Variable | Sub-category | Italy (N = 2033) | Sweden (N = 2121) |
| Likelihood | Epidemics | 96,41 | 98,59 |
|  | Floods | 97,34 | 99,62 |
|  | Drought | 97,69 | 99,53 |
|  | Wildfires | 97,59 | 99,72 |
|  | Earthquakes | 97,00 | 99,58 |
|  | Terror attacks | 96,06 | 98,68 |
|  | Domestic violence | 97,59 | 99,67 |
|  | Economic crises | 97,84 | 99,39 |
|  | Climate change | 97,98 | 99,48 |
| Damage to respondent | Epidemics | 96,36 | 95,85 |
|  | Floods | 97,44 | 98,07 |
|  | Drought | 97,39 | 99,01 |
|  | Wildfires | 97,39 | 98,68 |
|  | Earthquakes | 97,39 | 97,17 |
|  | Terror attacks | 95,97 | 96,79 |
|  | Domestic violence | 95,97 | 96,70 |
|  | Economic crises | 97,49 | 98,35 |
|  | Climate change | 97,29 | 98,30 |
| Damage to others in the country | Epidemics | 97,79 | 99,20 |
|  | Floods | 97,29 | 99,34 |
|  | Drought | 97,44 | 99,72 |
|  | Wildfires | 97,79 | 99,67 |
|  | Earthquakes | 97,59 | 98,77 |
|  | Terror attacks | 97,20 | 99,34 |
|  | Domestic violence | 96,66 | 98,87 |
|  | Economic crises | 97,88 | 99,62 |
|  | Climate change | 97,59 | 99,39 |
| Authorities' preparedness | Epidemics | 97,49 | 99,43 |
|  | Floods | 97,25 | 98,92 |
|  | Drought | 97,20 | 99,06 |
|  | Wildfires | 97,44 | 99,62 |
|  | Earthquakes | 97,39 | 98,59 |
|  | Terror attacks | 96,80 | 99,29 |
|  | Domestic violence | 96,56 | 98,16 |
|  | Economic crises | 97,54 | 99,06 |
|  | Climate change | 97,59 | 98,87 |
| Respondent's preparedness | Epidemics | 96,95 | 99,10 |
|  | Floods | 96,90 | 99,29 |
|  | Drought | 97,20 | 99,62 |
|  | Wildfires | 96,70 | 99,20 |
|  | Earthquakes | 96,90 | 99,29 |
|  | Terror attacks | 96,75 | 98,96 |
|  | Domestic violence | 95,38 | 97,45 |
|  | Economic crises | 97,00 | 99,25 |
|  | Climate change | 96,75 | 98,40 |
| Authorities' knowledge | Epidemics | 97,74 | 99,15 |
|  | Floods | 97,20 | 98,68 |
|  | Drought | 97,15 | 98,77 |
|  | Wildfires | 97,39 | 99,34 |
|  | Earthquakes | 97,39 | 97,97 |
|  | Terror attacks | 96,61 | 99,10 |
|  | Domestic violence | 96,41 | 98,21 |
|  | Economic crises | 97,20 | 98,96 |
|  | Climate change | 97,00 | 98,59 |
| Respondent's knowledge | Epidemics | 98,03 | 99,10 |
|  | Floods | 98,13 | 99,39 |
|  | Drought | 97,93 | 99,34 |
|  | Wildfires | 97,98 | 99,53 |
|  | Earthquakes | 97,88 | 99,39 |
|  | Terror attacks | 97,59 | 99,15 |
|  | Domestic violence | 97,20 | 98,49 |
|  | Economic crises | 97,88 | 99,29 |
|  | Climate change | 97,84 | 98,96 |
| Respondent's experience | Epidemics | 96,36 | 98,68 |
|  | Floods | 96,85 | 98,92 |
|  | Drought | 96,41 | 99,01 |
|  | Wildfires | 96,36 | 99,53 |
|  | Earthquakes | 96,36 | 99,58 |
|  | Terror attacks | 96,36 | 99,39 |
|  | Domestic violence | 96,46 | 99,81 |
|  | Economic crises | 96,11 | 98,68 |
|  | Climate change | 95,87 | 96,70 |
| Socio-demographics | Age | 100,00 | 100,00 |
|  | Gender | 100,00 | 100,00 |
|  | Education | 86,13 | 99,48 |
|  | Income | 97,79 | 99,10 |
|  | Employment | 96,56 | 99,48 |
|  | Employment sector | 75,36 | 85,20 |
|  | Political view | 89,23 | 98,07 |
